# Vision Restoration by Optogenetic Therapy and Developments Toward Sonogenetic Therapy

**DOI:** 10.1167/tvst.11.1.18

**Published:** 2022-01-13

**Authors:** Matthieu Provansal, Katia Marazova, José Alain Sahel, Serge Picaud

**Affiliations:** 1 Sorbonne Université, INSERM, CNRS, Institut de la Vision, Paris, France; 2 Department of Ophthalmology, The University of Pittsburgh School of Medicine, Pittsburgh, PA, USA; 3 Centre Hospitalier National d'Ophtalmologie des XV-XX, Paris, France

**Keywords:** vision, restoration, optogenetic, sonogenetic

## Abstract

After revolutionizing neuroscience, optogenetic therapy has entered successfully in clinical trials for restoring vision to blind people with degenerative eye diseases, such as retinitis pigmentosa. These clinical trials still have to evaluate the visual acuity achieved by patients and to determine if it reaches its theoretical limit extrapolated from ex vivo experiments. Different strategies are developed in parallel to reduce required light levels and improve information processing by targeting various cell types. For patients with vision loss due to optic atrophy, as in the case of glaucoma, optogenetic cortical stimulation is hampered by light absorption and scattering by the brain tissue. By contrast, ultrasound waves can diffuse widely through the dura mater and the brain tissue as indicated by ultrasound imaging. Based on our recent results in rodents, we propose the sonogenetic therapy relying on activation of the mechanosensitive channel as a very promising vision restoration strategy with a suitable spatiotemporal resolution. Genomic approaches may thus provide efficient brain machine interfaces for sight restoration.

## Introduction

Optogenetics is a recent technique to control or to monitor neural activity with light that can be achieved by the genetic introduction of light-sensitive proteins into the targeted cells.^1^^–^^3^ This concept of optogenetics has followed the initial discovery of optogenetic actuators like channelrhodopsin (ChR),^4^^–^^6^ halorhodopsin,^7^ and archaerhodopsin (Arch),^8^ which can modify the membrane potential of excitable cells and thus the activity of neurons. Similarly, sonogenetic therapy has been proposed to control neuronal activity by ultrasound waves following the genetic expression of an ultrasound-sensitive protein in neurons.^9^^,^^10^ Although optogenetics has already revolutionized the field of neurosciences, sonogenetic neuromodulation has only recently emerged. This review will focus on optogenetic and sonogenetic therapies as approaches for vision restoration in blind people. Optogenetic therapy was recently assessed in a blind patient, providing the first evidence for partial functional recovery of vision through this novel strategy,^11^ whereas sonogenetic therapy was only tested in rodents.^12^ However, both therapies offer great hopes for restoring some useful sight addressing different types of blindness.

### Optogenetic Vision Restoration at Retinal Level

In the past 2 decades, retinal prostheses have made great steps ahead from the perception of light to rendering the possibility to read. The retinal prosthesis PRIMA (Pixium Vision, Paris, France) provides today the best restored visual acuity in blind patients. This retinal prosthesis has been tested in patients affected by dry age-related macular degeneration allowing a prosthetic visual acuity between 20/460 and 20/565 in 4 patients with the ability to fuse the central infrared artificial vision and the natural peripheral vision.^13^^,^^14^ Despite potential improvements in this technology, it will be very difficult to reach a single cell resolution. By contrast, optogenetic therapy could allow a cellular resolution by rendering residual neurons in the retina sensitive to light. This alternative solution was first proposed by the team of Zhao Pan. They first showed that channelrhodopsin2 (ChR2) can be expressed in the retinal ganglion cells of *rd1* mouse after the complete loss of photoreceptors, allowing retinal ganglion cells to respond to light.^15^ Studies in dystrophic Royal College of Surgeons (RCS) rats also confirmed that light sensitivity can be restored through expression of ChR2 in retinal ganglion cells after complete photoreceptor degeneration.^16^^,^^17^ Subsequently, this approach was applied to marmoset retina with success^18^ and immediately translated to the clinical trial NCT02556736. No result has yet been published since the project started in 2015.

Following these initial studies, we have evaluated the possibility to express the microbial opsin with improved light sensitivity CatCh^19^ in retinal ganglion cells of non-human primates and demonstrated the advantages of a selective promoter.^20^ This optogenetic tool could potentially restore a theoretical visual acuity of 20/72.^21^ However, because microbial opsins require very high light intensities, we were concerned about using either ChR2 or its derived protein, CaTCh, which are both blue light sensitive. Indeed, we recently showed that neurons, such as photoreceptors, can be destroyed by blue light, likely due to the blue light absorption of porphyrins in their mitochondria.^22^ We therefore assessed the efficacy of two different red-shifted opsins, ReachR and ChrimsonR, to activate retinal ganglion cells.^23^^,^^24^ We finally opted for ChrimsonR, which is the most red-shifted opsin^25^ and selected the optimal AAV2-7m8 viral vector to optimize its expression in primates.^24^ Surprisingly, our study showed that the highest efficacy and likely highest protein expression was obtained for the fused protein with a red fluorescent reporter gene, ChrimsonR-tdTomato.^24^ We then demonstrated that in vivo optogenetic activation of retinal ganglion cells expressing ChrimsonR-tdTomato can activate the primate visual cortex,^26^ whereas others reported the in vivo activation of the primate retinal ganglion cells at the retinal level.^27^ Following these non-human primate studies, this strategy was brought into the clinical trial (NCT03326336) aiming at evaluating the safety and tolerability of ChrimsonR-tdTomato delivered by the AAV2-7m8 vector in subjects with retinitis pigmentosa. It has recently been shown that, after 14 years of blindness, a patient affected by retinitis pigmentosa recovered some partial vision.^11^ More patients participating in this clinical trial were now found to have some partially restored vision with this optogenetic strategy relying on the GS030 product from the company GenSight Biologics. Patients are wearing goggles to convert the visual scene into a 600 nm image using an asynchronous camera, which enabled us to model at a millisecond precision the activity of the retinal ganglion cells.^28^

Others have similarly created an image converter^29^ to enter into a clinical trial (NCT04278131) with another microbial opsin named Chronos,^25^ a strategy developed by the company Bionic Sight LLC. Another approach has combined several microbial opsins (ChR2, ReaCh, and C1V1) to generate the multicharacteristic opsin (MCO1) with a white light sensitivity and apparently the need for lower light levels.^30^^–^^32^ This approach was assessed in a dog^32^ prior to the recent development of a clinical trial with the company Nanoscope Therapeutics (NCT04945772).

Retinal ganglion cells are characterized by a large anatomic and functional diversity resulting in different light responses, which cannot be reproduced simultaneously and individually in each cell type by optogenetics. All retinal ganglion cells become ON cell types indifferently from their original cell types. To offer more variability, several strategies were proposed to target cells at a higher level in the retinal information processing sequence. For instance, bipolar cells were targeted to express ChR2 using a specific promoter^33^ through adeno-associated viral vector (AAV) delivery.^34^ We^35^ and others^36^ observed that optogenetic activation of bipolar cells could restore the ON and OFF responses in retinal ganglion cells using ChR2 and CatCh, respectively. The optimal strategy would be to reactivate “dormant” cone photoreceptors when they remain despite a loss of their natural photosensitivity.^37^^,^^38^ Complex information processing can be restored in such conditions but a limited number of patients affected by retinal dystrophies retain such dormant non-photosensitive photoreceptors.^37^

As microbial opsins require very high light intensities and they could potentially induce a negative immune response, various laboratories have expressed mammalian opsin ectopically to restore vision. Melanopsin was the first opsin to be targeted in retinal ganglion cells because this opsin is already present in a small population of intrinsically photosensitive retinal ganglion cells with non-visual functions.^39^ This opsin rendered all transfected retinal ganglion cells intrinsically sensitive to light but such melanopsin-expressing cells acquired light responses similar to those of intrinsically photosensitive retinal ganglion cells that are lasting for minutes, a dynamic incompatible for vision. Then, rhodopsin was expressed in residual neurons of the inner retina providing light responses at very low light levels but with a light onset developing over seconds.^40^^,^^41^ Cone opsin provided faster kinetics as cone response kinetics are also faster than those of rods.^42^ The later project will be developed by Novartis after its acquisition of the start-up, Vedere Bio. These surprising studies relying on such ectopically expressed mammalian opsins demonstrated that they can hijack metabolic pathways of other G proteins leading to ionic channel activation. Aside from natural mammalian opsins, ON-bipolar cells were also targeted for expressing Opto-GlumR6, a chimeric protein combining an opsin to the metabotropic glutamatergic receptor.^43^ This strategy is now followed by Novartis after acquiring the start-up Arcos Medical. Another engineered photoactivatable G-protein coupled receptor, SNAG-mGluR2, evoked OFF responses in retinal ganglion cells of the blind retina.^44^ Combined expression of two engineered proteins, SNAG-mGluR2 and LiGluR, generated both ON and OFF responses in different retinal ganglion cells.^44^

Finally, optogenetic gene therapy was also combined with cell therapy because photoreceptors generated from induced pluripotent stem cells are not yet able to generate active photosensitive outer segments. This strategy is appealing as it enabled us to transplant active photoreceptors in advanced states of retinal degeneration. Transplantation of engineered photoreceptors expressing inhibitory microbial opsins restored visual responses and behavior in blind animals.^45^^,^^46^

### Cortical Visual Restoration

For diseases leading to blindness following the loss of the optic nerve, cortical visual prostheses were developed in the 1960s by Brindley and Lewin with success in eliciting phosphenes in the visual field^47^ allowing Braille reading.^48^^,^^49^ However, the recovered sight tended to fade away in many patients. More recently, the novel cortical prosthesis, Orion from the company 2nd Sight, showed form recognition by sequential stimulation of the visual cortex with surface electrodes (500 µm) spaced every 2 mm.^50^ In parallel, other studies have shown that smaller currents are needed for stimulating the cortex in depth.^51^ As a consequence, the penetrating electrodes of the Utah arrays enabled primates to recognize letters in a more dynamic way.^52^ These experimental investigations were confirmed in a clinical trial with blind patients.^53^ Nonetheless, such penetrating electrodes have also been shown to lose their stimulation efficacy with time.^54^ These results on cortical visual prostheses have justified the search for a distant non-contact neuronal activation of cortical neurons.

Optogenetic therapy appeared as an obvious non-contact alternative for the distant activation of cortical neurons from the brain surface. Expression of a microbial opsin in the visual cortex was found to activate cortical neurons even in the cortex depth following surface illumination of the brain.^55^^–^^57^ In non-human primates, this cortical activation produced visual saccades toward the corresponding point in the visual field^55^^,^^56^^,^^58^ and was able to elicit activity in other visual areas as indicated by functional magnetic resonance imaging (fMRI).^58^ More subtle changes were described, such as an increased sensitivity to the stimulus orientation, only when the optogenetic stimulation was applied on a column with an orientation selectivity close to that of the stimulus.^59^^,^^60^ Furthermore, optogenetic activation of single ocular dominance columns generated preferential activation of nearby same-eye columns.^60^ Neuronal inhibition using ArchT opsin led instead to the partial suppression of visually-evoked responses and shifted the psychometric curves associated with the discrimination of visual stimuli based on their luminance.^61^ Again, the psychometric curves were shifted only when the location of the visual stimuli matched the receptive field of the stimulation.

For future clinical applications, it should be considered that deep brain optogenetic stimulation is difficult due to light absorption and scattering in the brain tissue,^62^ thus rendering optogenetic applications to non-human primates uncommon compared with rodents.^63^ In addition, complex visual tasks require at least 600 pixels, as demonstrated for locomotion, face recognition, and text reading.^64^^–^^66^ A pioneering study demonstrated the feasibility to use a chronically implanted surface LED array as a light source in nonhuman primates.^61^ Other investigators have proposed invasive light guide or penetrating LED arrays.^67^^–^^70^ These optical devices become highly invasive losing thereby the promise of optogenetics for distant non-contact activation of neurons.

In a recent study, we have proposed to use ultrasound waves instead of light to activate cortical neurons following targeted expression of a mechanosensitive ionic channel. This strategy of ultrasound activation of neurons was named sonogenetics, as it relies on the genetic expression of a mechanosensitive protein coupled to the ultrasound stimulation. Ultrasound waves are well known to propagate into the tissue depth and even deep into the non-human primate visual cortex, as it has been demonstrated in our study by brain cortical functional ultrasound imaging.^71^ Some neurons can be naturally sensitive to ultrasound waves and ultrasound neuromodulation was developed by different laboratories.^72^^–^^78^ This ultrasound sensitivity appears to rely on different proteins from the two-pore-domain potassium channel family expressed in neurons,^79^ namely the MEC-4 ionic channel in Caenorhabditis elegans^80^ and the mechanosensitive ionic channel Piezo1.^81^ Unfortunately, these ultrasound stimulations can generate refractory periods^78^ and hemorrhages due to the required high ultrasound energies.^82^ To reduce these unwanted effects, various proteins have been expressed ectopically in neurons to render them ultrasound sensitive. These ultrasound sensitive proteins include the auditory-sensing Prestin protein,^83^^,^^84^ the TRP-4 ionic channel,^85^ the TRPV1 ionic channel,^86^ and the mechanosensitive large conductance ionic channel MscL.^87^^–^^89^ All the in vivo studies using these proteins showed temporal resolution requiring several hundred milliseconds to a second for developing the response, which is too slow for visual restoration.^83^^–^^86^^,^^89^ We therefore investigated if a mechanosensitive protein can generate a sonogenetic activation with a spatiotemporal resolution compatible with visual restoration. We have shown that the G22S mutated version of the MscL ionic channel displaying an increased sensitivity to ultrasound stimulation can be reliably expressed in neurons of the visual cortex providing a millisecond temporal resolution and a spatial resolution in the 100 µm range when applying 15 MHz ultrasound waves at safe energies.^12^ We have also observed in a behavioral test that the ultrasound sonogenetic activation of the visual cortex is perceived as a light flash.^12^ These original results provide the first evidence that sonogenetic therapy could provide a safe strategy for restoring partial sight in blind patients following optic atrophy in diseases like glaucoma or diabetic retinopathy. Sonogenetic therapy may thus become a brain machine interface for restoring vision and other neuronal applications with a stimulation of distant deep brain structure realized without contact from a device standing above the dura mater following a local AAV injection to target the MscL expression in a selected neuronal population based on a specific cell promoter.

## Conclusions

Genomic strategies based on gene therapy expressing ectopically photosensitive and mechanosensitive ionic channels may provide high resolution brain-machine interfaces. Optogenetics is already in clinical trials with results demonstrating restored partial vision in patients affected by retinitis pigmentosa. Further analyses are needed to assess if patients can reach the theoretical visual acuity (20/72) estimated in ex vivo studies on the non-human primate retina. The results of ongoing clinical trials will thus decide if optogenetics can provide a convincing alternative to retinal prostheses for restoring vision. Similarly, sonogenetic therapy offers an alternative to cortical prostheses for a deep non-contact cortical stimulation from above the dura mater. Further studies are needed to evaluate the safety and efficacy of this strategy prior to launching clinical trials. Despite these current limitations, the results of our study suggest that sonogenetics holds great hope for a novel generation of brain machine interface for cortical visual restoration and other neurological applications.
